# Marked increases in mucociliary clearance produced by synergistic secretory agonists or inhibition of the epithelial sodium channel

**DOI:** 10.1038/srep36806

**Published:** 2016-11-10

**Authors:** Nam Soo Joo, Jin Hyeok Jeong, Hyung-Ju Cho, Jeffrey J. Wine

**Affiliations:** 1 The Cystic Fibrosis Research Laboratory, Stanford University, Stanford, CA 94305-2130, USA; 2 Department of Otolaryngology-Head and Neck Surgery, Hanyang University School of Medicine, Seoul, Korea; 3 Department of Otorhinolaryngology, Yonsei University, Seoul, Korea

## Abstract

Mucociliary clearance (MCC) is a critical host innate defense mechanism in airways, and it is impaired in cystic fibrosis (CF) and other obstructive lung diseases. Epithelial fluid secretion and absorption modify MCC velocity (MCCV). We tested the hypotheses that inhibiting fluid absorption accelerates MCCV, whereas inhibiting fluid secretion decelerates it. In airways, ENaC is mainly responsible for fluid absorption, while anion channels, including CFTR and Ca^2+^-activated chloride channels mediate anion/fluid secretion. MCCV was increased by the cAMP-elevating agonists, forskolin or isoproterenol (10 μM) and by the Ca^2+^-elevating agonist, carbachol (0.3 μM). The CFTR-selective inhibitor, CFTR_inh_-172, modestly reduced MCCV-increases induced by forskolin or isoproterenol but not increases induced by carbachol. The ENaC inhibitor benzamil increased basal MCCV as well as MCCV increases produced by forskolin or carbachol. MCC velocity was most dramatically accelerated by the synergistic combination of forskolin and carbachol, which produced near-maximal clearance rates regardless of prior treatment with CFTR or ENaC inhibitors. In CF airways, where CFTR-mediated secretion (and possibly synergistic MCC) is lost, ENaC inhibition via exogenous agents may provide therapeutic benefit, as has long been proposed.

Airway mucociliary clearance (MCC) is a critical host innate defense mechanism in airways and is impaired in airway diseases such as cystic fibrosis (CF)^1^,^2^, chronic obstructive pulmonary disease (COPD)^3^, primary ciliary dyskinesia (PCD)^4^, chronic rhinosinusitis (CRS)^5^, and asthma^6^. Mucociliary clearance depends upon mucin and fluid secretion. For airway clearance, MUC5B is the most critical mucin^7^. MUC5B originates from mucous cells in airway submucosal glands and in club cells^8^. Fluid, including critical ions and macromolecules that influence mucus rheology and its ability to inhibit microbial growth, is secreted by gland serous cells and surface epithelia, which depend upon the apical anion channels, cystic fibrosis transmembrane conductance regulator (CFTR) and Ca^2+^-activated chloride channels (CaCCs) to provide exit pathways for anion efflux onto the airway luminal surface. Fluid depth is also controlled by fluid absorption from airway surface epithelia via the epithelial sodium channel (ENaC). This is also critical, as shown by the mucus obstruction observed in transgenic mice overexpressing ENaC^9^.

Optimal airway mucociliary clearance depends upon the speed and effectiveness of ciliary beating, the depth and rheological properties of the mucus, and structurally intact (e.g. not bronchiectatic) airways. Of these, the rheological and antimicrobial properties of mucus are most critically affected in early CF (prior to chronic infection) by the loss of CFTR-mediated anion (particularly HCO_3_^−^) and fluid secretion^10^,^11^. Mucus clearance occurs autonomously, but its rate is normally regulated by parasympathetic (vagal) innervation.

Ballard and colleagues pioneered the use of *ex vivo* pig tracheas for studies of MCC^12^,^13^, and we extended that work to the *ex vivo* ferret trachea^14^. In our work we measured basal and agonist-stimulated MCC velocities (MCCV) in response to agonists and ion transport inhibitors whose effects on mucus secretion by ferret submucosal glands had previously been quantified^15^. One result was that combinations of threshold levels of agonists that elevated [cAMP]_i_ with those that elevated [Ca^2+^]_i_ produced synergistic increases in MCCV. Another was that the Na^+^/K^+^/2Cl^-^ cotransporter (NKCC) inhibitor bumetanide reduced or abolished agonist-stimulated MCCV, whereas HCO_3_^−^-free solutions did not. Of particular interest, agonists that elevated [cAMP]_i_ increased MCC much more effectively than expected from their relatively small effects on gland mucus secretion rates. Finally, bumetanide almost completely inhibited [cAMP]_i_-stimulated MCC, but had a smaller effect on gland secretion^14^.

In the present study, we asked if the specific CFTR inhibitor CFTR_inh_-172 would affect MCC in the *ex vivo* ferret trachea in the hope that inhibition of CFTR might approximate a pharmacological model of MCC in a CF trachea. CF ferrets have been made, but their airways are poorly developed at birth and mortality is presently too high to permit their use in experiments like ours. We also asked if the specific ENaC inhibitor benzamil would affect MCC in the *ex vivo* ferret trachea, based on extensive *in vivo* studies suggesting that inhibition of ENaC might increase MCC velocities^16^,^17^, and one *ex vivo* study in pig tracheas in which benzamil largely counteracted the decrease in MCCV observed with anion transport inhibitors^12^. We stimulated MCC using agents that elevated [cAMP]_i_ or [Ca^2+^]_i_. Finally, we also reexamined combinations of the two types of agonists using higher levels than those used previously.

Our results in this *ex vivo* system show that treatment with CFTR_inh_-172 slowed MCCV, but only when it had been stimulated with agents that elevate [cAMP]_i_ exclusively. When low levels (0.3 μM) carbachol were added to forskolin or isoproterenol, a synergistic increase in MCC occurred that appeared to be near maximal regardless of the prior treatment of the tissues. If this synergistic increase in MCCV occurs in human airways, methods to activate it could prove to be therapeutic in some diseases. However, synergy may be lost or blunted in CF airways, as is the synergistic increase in gland mucus secretion^18^,^19^,^20^. Fortunately, we also found that inhibition of ENaC speeds MCCV, buttressing the argument for the use of exogenous ENaC inhibitors in airways diseases with impaired MCC^21^ (and references therein).

## Results

### Setup and protocols for studies of MCC in *ex vivo* ferret tracheas

**Figure 1:**
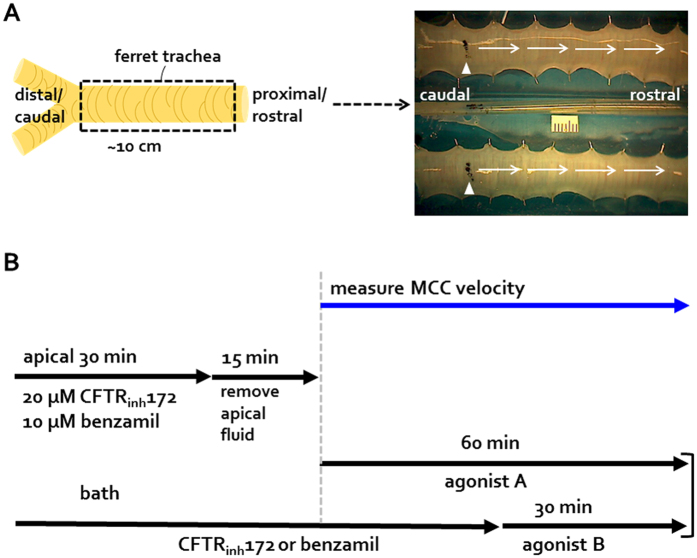
Ferret MCC set up and drug treatment protocol. Dissected and opened ferret tracheae were mounted on Sylgard platforms using insect pins and tracking particles (arrowheads) were placed onto caudal/distal region of tracheae. MCC was initiated by adding an agonist (10 μM forskolin or 0.3 μM carbachol) in the presence of absence of an ion channel inhibitor to the bath (**A**). Tracheae were pretreated with either 20 μM CFTR_inh_-172 or 10 μM benzamil both apically and basolaterally for 30 min (0.1% DMSO was the vehicle control). After removing the apical fluid and refreshing the baths, tracheas were stabilized for 15 min before adding agonists ± inhibitors to the baths. MCC velocity was measured in the presence of agonist A ± an inhibitor for 30 min, then with agonist A + agonist B in the absence of an inhibitor for additional 30 min.

Figure 1A shows a schematic of the tracheal preparation and one frame of a series of digital images used to track particle movement. Tracheas from two ferrets were pinned out in a sealed humidified chamber with a shared atmosphere but separate baths. In each experiment one trachea was designated as the control and the other as the experimental preparation. Paired experiments were preferred because of variations among batches of ferret tracheas. Ink particles were deposited onto the surface via an indwelling pipet and their transport followed with digital imaging (Supplementary Figure S. 1). Figure 1B diagrams the protocol. Full details are given in ref. 14 and methods. Briefly, the experimental trachea was treated with channel inhibitors apically and basolaterally for 30 min while the control tracheas were treated with vehicle. The apical fluid was then removed, the baths refreshed, and after a 15 min period to allow MCC to stabilize to a basal level, agonists were added basolaterally (defined as time 0) and the 60 min measuring period begun. After 30 min the inhibitors were removed from the bath and a second agonist was added to assess possible synergy (see Supplementary MCC video). Basal MCC velocities were measured at T0-T1 (before agonist effects). Stimulated MCC was measured at 5 min intervals from T5 to T60. Experiments of a given type were done together (typically 2–3 paired experiments per day) on the same day the tissues were received unless otherwise noted.

### Basal MCCV was increased by benzamil and by overnight storage of tissue

**Figure 2:**
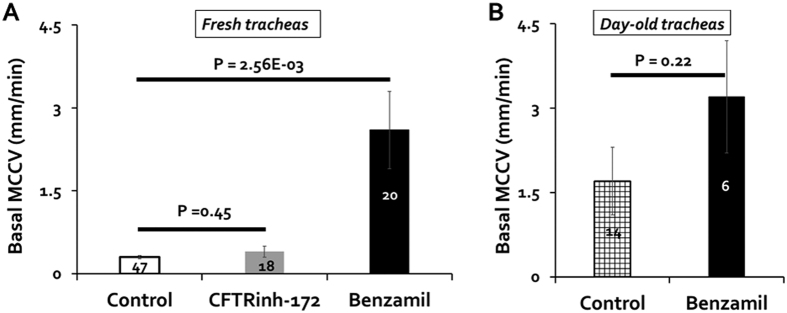
Benzamil increased basal MCCV. Basal MCCV was measured at T0-T1 prior to the onset of agonist effects. (**A**) Basal MCCV in three groups: control (open column), CFTR_inh_-172 pretreatment (gray column) and benzamil pretreatment (black column). Number of tracheas tested is shown for each condition. Control is based on all experiments with forskolin or carbachol agonists but no inhibitor pretreatment. Benzamil produced a significant increase in basal MCCV compared to control (P = 0.0026) or CFTR_inh_-172 (P = 0.0029) conditions. (**B**) Summary of basal MCCV in control and benzamil (Bz)-treated day-old tracheas (data from T0-T1 0.3 μM carbachol + 10 μM benzamil). P values came from Student *t*-tests. Day-old tracheas were used for comparison purposes only in Figs 2 and 5. All other experiments were done with fresh ferret tracheas.

Benzamil increased basal MCCV. We did not explicitly design our experiments to measure basal MCCV. However, by comparing experimental and control MCCV in the T0-T1 period, we were able to assess basal MCC at the end of the 15 min stabilization period before the agonist effects started. In Fig. 2A (and see Supplementary MCC video) we compare mean basal MCCV for 47 control tracheas pooled from all experiments without inhibitor pretreatment, 18 tracheas pretreated with 20 μM CFTR_inh_-172, and 20 tracheas pretreated with 10 μM benzamil. As shown, benzamil increased the basal MCCV (mean control basal *vs*. basal with benzamil in mm/min = 0.3 ± 0.03 and 2.6 ± 0.7, P = 0.003, Fig. 2A). By contrast, we observed no effect of CFTR_inh_-172 on basal MCCV measured in the same way (mean control basal and basal with CFTR_inh_-172 in mm/min = 0.3 mm ± 0.03 and 0.4 ± 0.1 P = 0.45, Fig. 2A).

Tissues from human lung transplant recipients and from CF animal models frequently need to be shipped overnight from their sources to distant investigators. Therefore, it is important to assess changes that might occur in such tissues intended for use in *ex vivo* experiments. Mucus secretion rates from submucosal glands in day-old human and pig tissues are equivalent to those of same-day tissues^22^,^23^. To determine if MCCV was also maintained in day-old tissues, we compared basal MCCV of day old tracheas vs. fresh tissues (Fig. 2B). Surprisingly, mean basal MCCV was significantly *faster* in day-old tissues: (Fig. 2B, grid column: 1.7 ± 0.6, P = 0.024 *vs*. fresh control). When a physiological process improves with time *ex vivo* it suggests the waning of an inhibitory process. Because fluid absorption via ENaC is expected to slow MCCV, reduced ENaC-mediated absorption in day-old tissues might be responsible for the MCCV increase. Benzamil did increase basal MCCV in day-old tissues Fig. 2B, but not significantly *vs*. fresh basal (P = 0.22). We examine the interaction between benzamil effects and tissue age again in the section on carbachol-stimulated MCCV. (Note that the fresh vs. day-old comparisons can’t be done with paired tissues).

### CFTR_inh_-172 reduced MCCV stimulated by forskolin or isoproterenol

**Figure 3:**
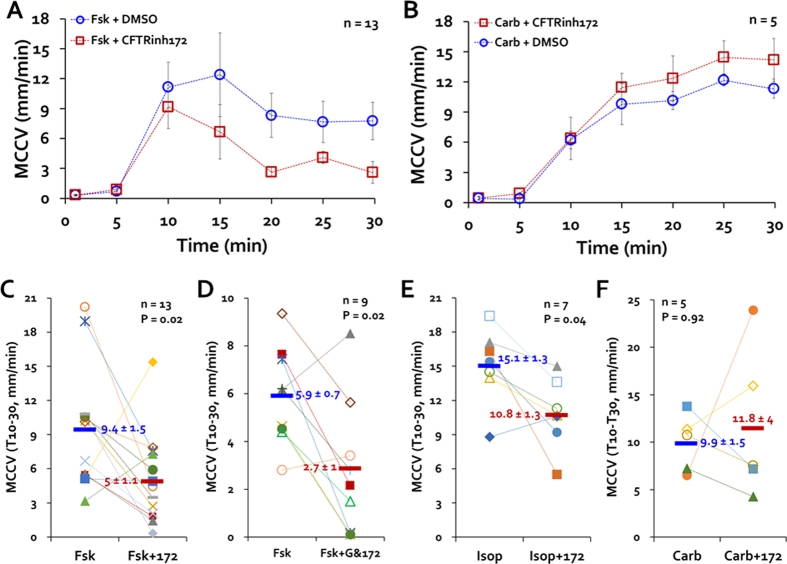
CFTR_inh_-172 reduced MCCV-stimulated by forskolin, but not by carbachol. (**A,B**) Time course (T1-T30) of ferret MCC-initiated by 10 μM forskolin (Fsk, **A**) or 0.3 μM carbachol (Carb, **B**) ± 20 μM CFTR_inh_-172. (**C–F**) Summary data for CFTR inhibitors. Each point is the average T10-T30 MCCV of an individual trachea in response to the agonists shown ± the inhibitors shown. Horizontal bars show average for each set of experiments; n values and P values (by Mann-Whitney U tests) are in upper right or left. Abbreviations: Fsk = 10 μM Fsk; Isop = 10 μM isoproterenol; Carb = 0.3 μM carbachol; 172 = 20 μM CFTR_inh_-172; G = 50 μM GlyH101.

MCC velocities are increased by forskolin^14^, which directly activates adenylate cyclase to increase [cAMP]_i_. When we added 10 μM forskolin, little change in average MCCV was observed at T0 and T5, (average velocities < 1 mm/min), but velocities rose sharply to >10 mm/min from T10-T15, and then declined to a stable value of ~8 mm/min for T20-T30 (Fig. 3A, blue circles). When stimulated after pretreatment with and in the presence of CFTR_inh_-172, MCCV was decreased significantly at later but not earlier time points (Fig. 3A, red squares). Figure 3C shows that the mean forskolin-stimulated T10-T30 MCCV was ~halved by CFTR_inh_-172 (means in mm/min): control 9.4 ± 1.5 *vs*. CFTR_inh_-172 treated 5.0 ± 1.1, (P = 0.024, n = 13). When 50 μM of GlyH101, a less specific channel inhibitor, was combined with 20 μM CFTR_inh_-172 in the forskolin-stimulation protocol (Fig. 3D), the combined inhibitors were not significantly more effective than CFTR_inh_-172 alone (% inhibition: CFTR_inh_-172, 25.3 ± 25.6 *vs*. combined, 51.8% ± 17.9, P = 0.4). (However, note that the single vs. combined inhibitors were not compared in paired experiments). Figure 3E shows that similar results were obtained when 10 μM isoproterenol, a β-adrenergic agonist, was used instead of forskolin in this protocol.

### MCC stimulated by low-dose carbachol was not inhibited by CFTR_inh_-172

Figure 3B shows that MCCV was increased by 0.3 μM carbachol to about the same extent as they were by 10 μM forskolin or isoproterenol. However, in contrast with what was seen with the [cAMP]_i_ -elevating agonists, 20 μM CFTR_inh_-172 did not inhibit the response to carbachol. Averaged MCCV T10-T30 in mm/min was: control, 9.9 ± 1.5 *vs*. CFTR_inh_-172-treated, 11.8 ± 4 (P = 0.92, n = 5 each) (Fig. 3F).

### Benzamil increased forskolin- or carbachol-induced MCC

**Figure 4:**
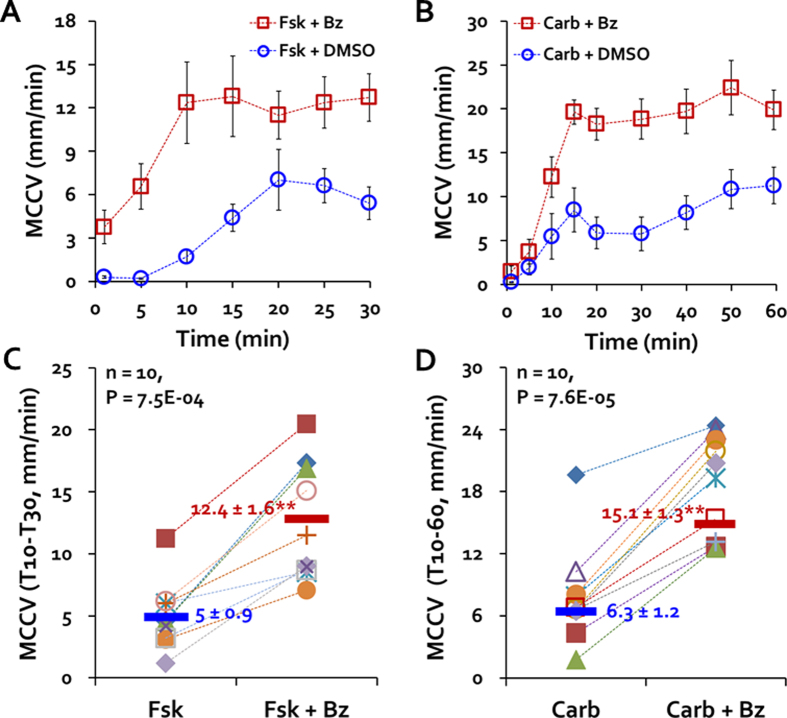
Benzamil increased MCC-stimulated by forskolin or by carbachol. (**A,B**) Time courses of changes in ferret MCCV-initiated by 10 μM forskolin (Fsk, 4A) or by 0.3 μM carbachol (Carb, 4B) ± 10 μM benzamil (Bz). (**C,D**) Summary data. Each point is the average T10-T30 MCCV for an individual trachea in response to agonist alone or agonist + benzamil. P values came from Mann-Whitney U tests.

When 10 μM of the ENaC inhibitor benzamil^24^,^25^ was used in the MCC protocol, forskolin-stimulated MCCV was markedly increased compared to paired control tracheas (Fig. 4A and see Supplementary MCC video). The basal MCCV was significantly increased in the benzamil-treated tracheas (in mm/min, Fig. 4A, 0.3 ± 0.1 for controls and 3.8 ± 1.2, P < 0.001). The average T10-T30 MCCV in mm/min was 5.0 ± 0.9 for forskolin-stimulated controls, vs. 12.4 ± 1.6 for the benzamil-treated tracheas (P < 0.001, n = 10 each, Fig. 4C). Benzamil treatment also markedly increased carbachol-stimulated MCCV. In some systems carbachol induces transient responses. To see if MCCV would decline over time, we extended the measurement of MCCV for 6o min. Figure 4B shows that both the 0.3 μM carbachol-stimulation and its enhancement by benzamil were sustained for at least 60 min. The basal MCCV was significantly increased in the benzamil-treated tracheas (in mm/min, Fig. 4B, 0.3 ± 0.1 for controls and 1.4 ± 0.7, P < 0.023). The T10-T60 average MCCV was in mm/min = 6.3 ± 1.2 and was increased to 15.1 ± 1.3 with benzamil treatment (P < 0.0001, n = 10 paired tracheas, Fig. 4D).

### Carbachol stimulation was increased and benzamil effects were decreased in day-old tissues

**Figure 5:**
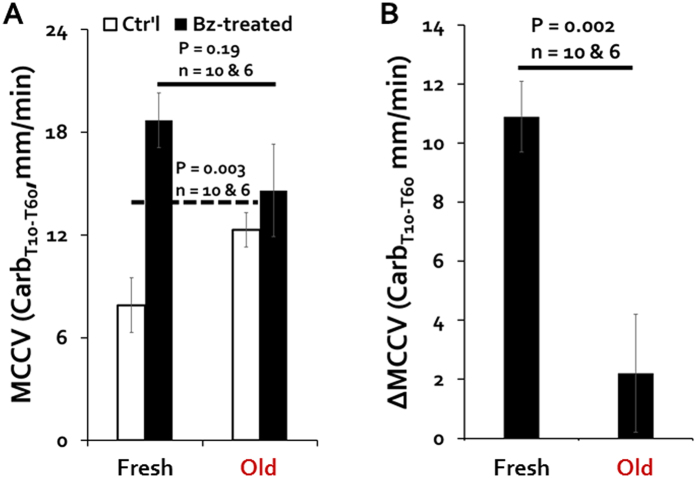
Decreased MCCV in day-old tracheas; evidence for a reduction in ENaC-mediated absorption. (**A**) Summary of carbachol-stimulated MCCV ± benzamil in fresh and old tracheas. (**B**) Deltas produced by benzamil on carbachol-stimulated MCCV in fresh *vs*. old tracheas—data taken from B and plotted separately for clarity. P values came from Mann-Whitney U tests.

We saw in Fig. 2 that basal MCCV was increased by either benzamil and by overnight storage. Here, we compare the effects of benzamil on MCC velocities stimulated by 0.3 μM carbachol in fresh and day-old tracheas (Fig. 5A). The mean T10-T60 MCCV produced by 0.3 μM carbachol was significantly faster in day-old vs fresh tracheas (Fig. 5A, P = 0.003, white bars). By contrast, both the absolute and the relative increases in MCCV produced by benzamil were smaller in day-old tracheas than in fresh ones (black bars). For clarity, Fig. 4B shows the deltas in MCCV produced by benzamil on carbachol-stimulated MCCV in fresh and day-old tissues. We interpret these data to mean that ENaC-mediated absorption by surface epithelia was reduced in day old tracheas.

### Synergy between 10 μM forskolin or isoproterenol + 0.3 μM carbachol stimulated MCCV to near maximum regardless of prior treatments with CFTR or ENaC inhibitors

**Figure 6:**
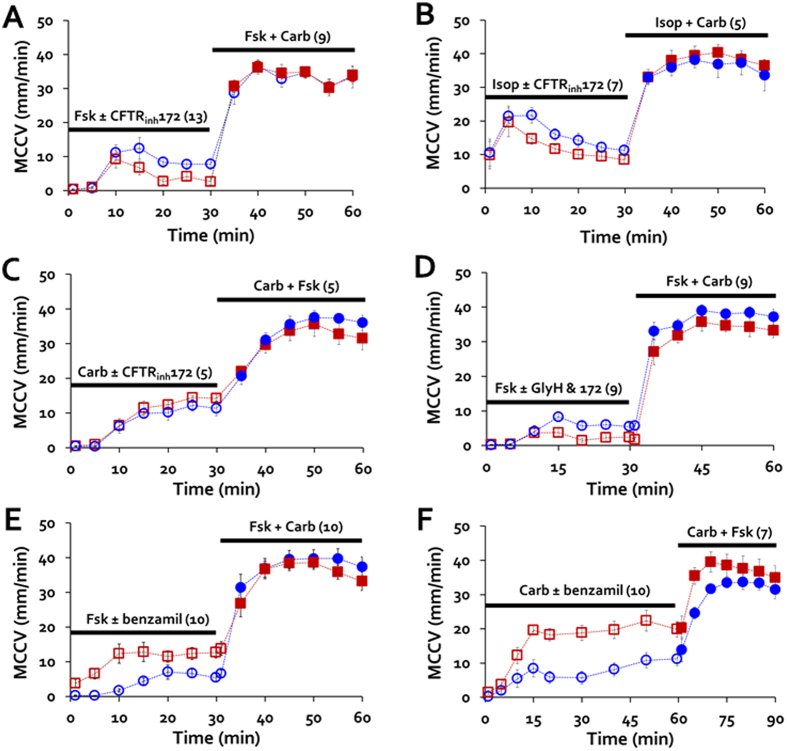
Marked increases in MCCV by combining cAMP and Ca^2+^ agonists. (**A–F**) Time courses of MCCV-initiated by single, followed by combined agonists ± inhibitors. Abbreviations are as in previous legends; the numbers of paired tracheas for each condition are shown in parentheses. Blue circles are agonists alone and red squares are an agonist + inhibitor(s). MCC velocities were markedly increased by combined agonists regardless of prior any inhibitor treatments or orders of drug additions.

**Figure 7:**
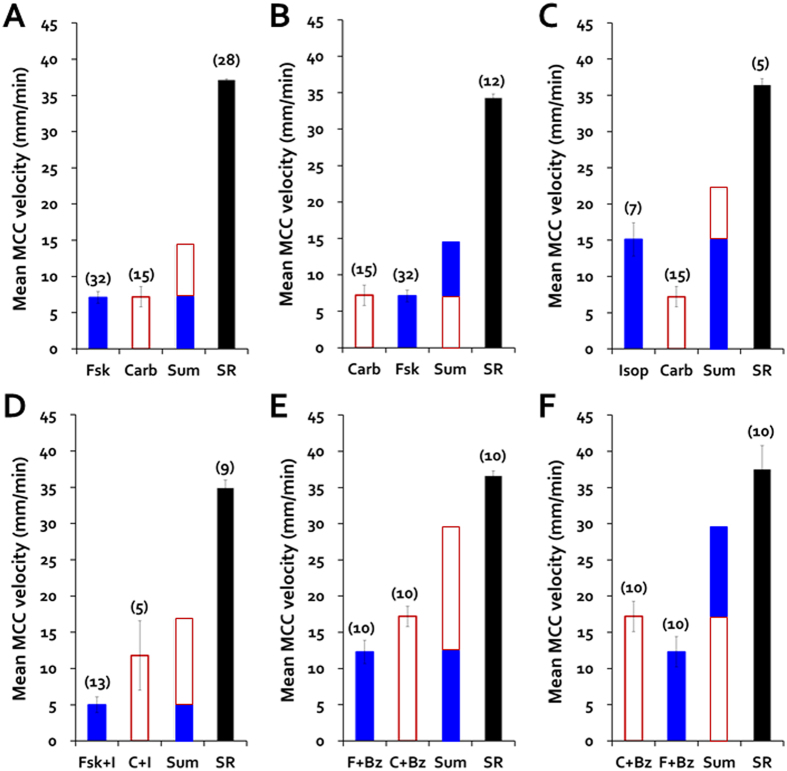
Summary of synergistic increases in MCCV by combining cAMP and Ca^2+^ agonists. **(A–F)** Data were pooled from the controls of all forskolin (Fsk) and carbachol (Carb) experiments. Columns in each panel show, from left to right, the responses to forskolin (blue bar), carbachol (red open bar), isoproterenol (Isop), or alone, then the arithmetic sum (Sum) of those two responses (hence there is no error bar in the Sum columns), and finally (black column) the actual synergistic response (SR) observed. The sample numbers are shown at the top of individual bars.

In each of the above experiments the inhibitors were removed after 30 (or 60) min and the complementary Ca^2+^ or cAMP agonist was added. In every case, MCCV increased markedly with the combined agonists (Fig. 6 and see Supplementary MCC video). Little difference was observed between tracheas that had been pretreated with CFTR or ENaC inhibitors and the controls, and the order of addition of the agonists did not seem to matter. Figure 7 illustrates that the increased MCCV was greater than the expected sum of the two agonists in every experimental condition. Note that all synergy conditions reach a similar level of MCCV, and that the difference between the summed responses and the synergy response is smallest for the experiments where benzamil was present (Fig. 7E,F).

**Figure 8:**
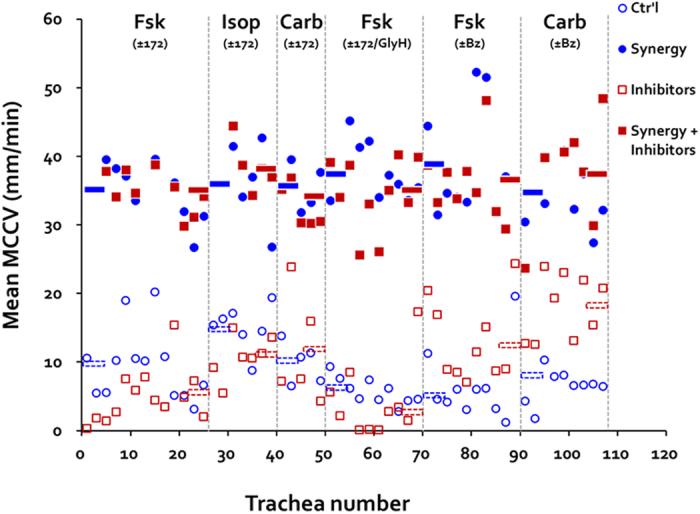
Summary of results across 12 conditions. Each point represents the average MCCV for a single trachea/condition. Open blue circles show T10-T30 MCCV for tracheas with the agonist shown; open red squares are in the presence of the inhibitors shown. Closed circles represent synergy conditions; closed circles show synergy in the presence of the inhibitors shown.

Figure 8 shows the mean T10-T30 values for each trachea after single agonist (blue open circles), after agonist + inhibitor (red open squares), and the mean T40-T60 (or T70-T90) values after the second agonist without (blue closed circles) or with (red closed squares) inhibitor pretreatment.

## Discussion

MCCV was increased by agents that increased either [cAMP]_i_ or [Ca^2+^]_i_. It was also increased by the ENaC inhibitor benzamil, which further increased MCCV when used in combination with either agonist. CFTR inhibitors modestly depressed MCCV stimulated by [cAMP]_i_ –elevating agents, but not basal MCCV nor that stimulated by [Ca^2+^]_i_ –elevating agents. By far the largest effects we observed were the increases in MCCV produced by combining [cAMP]_i_ and [Ca^2+^]_i_ –elevating agonists, which acted synergistically. The magnitude of MCCV produced by synergistic stimulation exceeded effects on gland fluid secretion seen in previous experiments^15^,^18^,^19^, and the effects appeared to nearly maximize MCCV, because similar levels were reached regardless of prior treatment of the tissues with agents that slowed or increased MCCV.

### Stimulated MCCV in this *in vitro* study was similar to ‘basal’ MCCV *in vivo*

The range of MCC velocities we observed in stimulated, *ex vivo* tracheas are comparable to those measured *in vivo*, where ‘basal’ (not deliberately stimulated) MCCV of 18.2 mm/min was approximately doubled by an anticholinesterase^26^. Species differ in their MCC velocities. Earlier reports of tracheal basal MCCV in rats^27^, dogs^28^, and cats^29^ were 14–22 mm/min. Fiberoptic bronchoscopy of Teflon micro discs in human subjects measured tracheal MCCV of ~20 mm/min with Vmax = 35 mm/min^30^. Computed tomography-based particle-tracking in anesthetized newborn pigs measured variable MCCV with Vmax of 20–25 mm/min^31^.

### Modest inhibition of MCCV by CFTR_inh_-172

The modest inhibition of forskolin-induced MCCV we observed after treatment with CFTR_inh_-172 is consistent with both incomplete inhibitions of CFTR and with CFTR-independent effects. Inhibition was CFTR-specific because carbachol-stimulated MCCV was not inhibited. Incomplete inhibition of CFTR by CFTR_inh_-172 is consistent with partial inhibition of observed in previous studies of CFTR-mediated I_sc_ in cultured ferret airway cells^32^ and with small effects on forskolin-mediated glandular secretion in multiple species (N. S. Joo, unpublished observations). However, CFTR-independent effects of forskolin on MCCV may also play a role—for example forskolin is known to increase ciliary beat frequency in some systems^33^,^34^,^35^, but also see^36^.

### Larger increase in MCCV by benzamil

By contrast with the modest effect of CFTR_inh_-172, the ENaC inhibitor benzamil markedly increased basal MCCV and MCCV stimulated by either forskolin or carbachol. We previously noted four components that influence MCCV: gland secretion, epithelial surface secretion, ciliary beat frequency, and epithelial sodium absorption^14^. We previously ruled out effects of ENaC inhibitors on gland secretion^37^, and although we did not measure ciliary beat frequency in the presence of benzamil, a prior report in pig tracheas saw no effect^12^. Thus, the most likely mechanism by which benzamil increased MCCV is decreased ENaC-mediated fluid absorption by surface epithelial cells.

What is the relevance of ENaC inhibition for mucus clearance in people and animals with cystic fibrosis? The possibility that ENaC is hyperactive in CF airways is controversial^38^,^39^,^40^,^41^,^42^,^43^. Thus, it is important to emphasize that increased MCCV following benzamil does not depend upon ENaC being overactive, so that using exogenous ENaC inhibitors to improve mucus clearance, which has long been advocated, would appear to be a valid approach if ENaC has any significant level of activity in the airways^21^,^44^,^45^,^46^,^47^. Practical issues are coupling long duration of action with absence of systemic effects. Therefore, our results support the continued development of safe, long-acting ENaC inhibitors^16^,^17^, as well as continued investigation of *endogenous* ENaC inhibition^25^,^45^,^48^,^49^,^50^. Evidence that ENaC-mediated I_sc_ by surface epithelia can be decreased by cholinergic stimulation^25^,^51^,^52^ suggests that ENaC inhibition might be a component of physiologically mediated increases in mucus clearance from the airways. If so, strategies might be devised to increase it.

### Biggest effects for synergy

By far the largest effects we observed resulted from the synergistic combination of forskolin with a low level of carbachol. In terms of the four components of MCC mentioned above, the synergy paradigm is known to increase gland secretion, but its effects on epithelial surface secretion and ciliary beat frequency have not been established. However, there is evidence across multiple species that the net effect of cholinergic stimulation is decreased ENaC-mediated Isc^25^,^51^,^52^, which will result in decreased fluid absorption. We have not yet determined if the addition of carbachol in the synergy paradigm also reduces ENaC-mediated Isc, but it is interesting that tissues pre-treated with benzamil did not show significantly faster MCCV in the synergy condition. While this could indicate a role for carbachol-stimulated inhibition of absorption, it is also possible that the benzamil effect had waned or MCCV was near-maximal.

The prior study of cholinergic inhibition of ENaC-mediated Isc used higher levels of cholinergic stimulation and so may not be comparable to the present studies^25^. On the other hand, some evidence in that work suggested that longer exposure to reduced levels of muscarinic agonists can lead to inhibition. In that light, the rise in MCC observed in the 30–60 min period after stimulation with carbachol (Fig. 4B, blue circles) is interesting and deserves further study.

What is the relevance of the synergy results for mucus clearance in people with cystic fibrosis? As stated, MCCV is driven in part by glandular mucus secretion, for which two kinds of synergy have been described. One, seen with low levels of [cAMP]_i_ and [Ca^2+^]_i_ –elevating agonists, is CFTR-dependent and is lost in CF humans^18^. This kind of synergy has been interpreted to represent increased driving force for anion exit through CFTR following activation of K^+^ channels, but increased activity of CFTR itself is also possible^53^. A second type, seen with higher levels of agonists, increases [Ca^2+^]_i_ and produces stimulation via non-CFTR pathways and so does not require CFTR^19^,^54^. For example, gland mucus secretion in CF pig glands was stimulated with 3 μM forskolin and 0.1 μM carbachol, but not by either agonist alone^19^. However, synergistic secretion via CaCC pathways was lower in CF pigs than in controls, indicating a remaining role for CFTR^19^. With strong cholinergic stimulation, abundant gland secretion is observed in CF humans and animals^19^,^20^,^22^,^55^, although it is statistically reduced. Synergy between [cAMP]_i_ and [Ca^2+^]_i_ –elevating agonists for gland mucus secretion and for MCCV is physiologically relevant because the predominant innervation of the airways is via the vagus and intrinsic airway neurons which use ACh and VIP as co-transmitters^56^ (and references therein). Thus, co-activation of these pathways, probably by transmitter concentrations far below those typically used in the laboratory, is the physiologically relevant stimulus.

### Limitations of this study

Our parametric studies of airway gland secretion and mucus clearance in ferrets were begun in anticipation of doing comparable work in CF ferrets, but to date it has not been practical to obtain sufficient CF ferret tracheas. As shown here, we failed to mimic CF by acutely inhibiting CFTR pharmacologically. Improvements in CF ferret survival^20^, conditional CFTR knockout ferrets, or greatly improved CFTR inhibitors are needed. CFTR inhibitors will need to be potent, because of evidence that even low levels of CFTR function are sufficient to stave off most CF symptoms^57^,^58^.

In our experiments with paired *ex vivo* ferret tracheas, basal MCCV dropped to near zero after a 15 min period without stimulation, as previously reported^14^. Basal MCCV in *ex vivo* tracheas is minimal and of questionable physiological relevance, because the tracheas are denervated and lack the airway surface liquid that would normally be transported from distal airways. Therefore, we did not study basal MCCV explicitly, but instead used the T0-T1 MCCV at the end of 15 min stabilization period prior to onset of agonist effects. The very low basal MCCV probably explains the lack of a CFTR_inh_-172 effect.

Carbachol-stimulated MCCV was increased in day old ferret tracheas and the delta to benzamil was decreased. We hypothesize that decreased ENaC activity is responsible for both phenomena, but because of the complexity of ENaC regulation^59^, we did not search for mechanisms or alternative explanations.

We did not measure changes in airway surface liquid (ASL) depth, pH, protein constituents, or ciliary beat frequency that might have resulted from the treatments and contributed to changes in MCCV^10^,^11^,^60^,^61^,^62^,^63^. Unusual effects can occur. For example, ferret tracheal submucosal gland mucus contains the proteases cathepsin S and H and their antiprotease cystatin C, and in CF ferrets the two proteases were increased more than the antiprotease, leading to an imbalance that inhibited ENaC–mediated fluid absorption and raised the height of the airway surface liquid (ASL) yet decreased MCCV^64^. Airway epithelia of CF ferrets absorbed fluid more slowly, but still became abnormally dehydrated with time. Analysis suggested that coupling of CFTR and ENaC activities was required for effective MCC and ASL height equilibration following volume challenge. It will be important to merge the approaches used by Evans *et al*.^64^ with the paradigm used here to more fully understand MCC in health and disease.

### Summary

MCCV of isolated ferret tracheas was accelerated by treatments expected to increase fluid secretion or decrease fluid absorption. The results suggest that MCCV will be reduced in CF, and support the long-standing proposal that inhibiting ENaC should be beneficial in CF—this does not require that ENaC be upregulated in CF. By far the largest increases in MCCV, to near-maximal values, were obtained by combining 10 μM forskolin and 0.3 μM carbachol. The synergism observed by adding such a small amount of carbachol (only 3% of the concentration usually used in MCCV experiments) was unexpected. It greatly exceeds synergistic effects observed previously on glandular fluid secretion^14^ and overwhelms decreases produced by CFTR_inh_-172 or increases produced by benzamil. Synergy is likely the normal condition *in vivo* (where agonists for both pathways are co-released by airway intrinsic neurons^56^,^65^); but it is unknown to what extent it persists in CF airways. Glandular mucus secretion rates are correlated with MCCV^12^,^14^,^64^, and synergy for that function is absent or reduced in CF depending upon level of agonists used^18^,^19^; but any remaining synergy might be exploited—indeed some treatments might already be taking advantage of these pathways. Further advances in answering these questions will require both *ex vivo* and *in vivo* experiments with CF animals (and eventually humans).

## Materials & Methods

### Airway tissue procurement

*Mustela Putorius* ferret tracheas of 6~36 months old were obtained 1–2 hr postmortem by pentobarbital sodium injection after acute experiments unrelated to our present study. Unless otherwise noted, experiments were conducted within 8 hr postmortem (n = 128). All protocols for handling animal tissues at Stanford were approved by Administrative Panel on Laboratory Animal Care (Stanford’s Institutional Animal Care and Use Committee: IACUC protocol#: 10048). Methods for ferret tissues were carried out in accordance with approved guidelines. Harvested tissues were placed in cold PhysioSol^TM^ (Hospira, IL) for transport to the laboratory and then transferred to ice-cold Krebs Ringer bicarbonate (KRB) buffer gassed with 95% O_2_ and 5% CO_2_ until the mucociliary clearance assays. The KRB buffer composition was 115 mM NaCl, 2.4 mM K_2_HPO_4_, 0.4 mM KH_2_PO_4_, 25 mM NaHCO_3_, 1.2 mM MgCl_2_, 1.2 mM CaCl_2_, and 10 mM Glucose (prepared pH 7.2 at room temperature and increased to pH 7.4 at 37 °C with a continuous supply of humidified 95% O_2_ and 5% CO_2_) adjusted to ~290 mosM with a Wescor vapor pressure osmometer. The Krebs buffer contained 1.0 μM indomethacin to minimize endogenously generated prostaglandins during tissue preparation. Tracheas with intact cartilage were dissected from other attached tissues in ice-cold KRB solution and then transferred to KRB at room temperature before mounting them onto MCC Sylgard platforms (Fig. 1 and Supplementary Figure S. 1).

### Measurement of mucociliary clearance velocity (MCCV)

Methods for measuring MCCV were described in detail previously^14^, but using a slightly modified chamber that minimized potential unequal hydration of surface epithelia (see Supplementary Figure 1). Briefly, each whole length trachea was mounted mucosal side up after the dorsal was cut open so that the serosal side of the tissue preparation was bathed in KRB buffer while the mucosa was exposed to the gas. The ends of the trachea were raised about 0.5 cm from the surface of the platform to prevent potential re-entry of the bath buffer (Supplementary Figure S. 1B). Two tracheal preparations were placed side-by-side in a sealed chamber and continuously gassed with fully humidified and warmed 95%/5% O_2_/CO_2_. The chamber/bath temperature was increased from room temperature to 37 °C over 10–15 min. A mm scale was centrally placed between the two tracheas (Figure 1A) and MCCV was measurement as the movement of Xerox ink toner particles placed on the ASL surface ~1.5 cm from the caudal end at 5 min intervals. Every 5 min, 4 images (at 20 second intervals) were automatically captured using an Aven zipScope digital camera and associated software (Ann Arbor, MI) and stored on a PC. Stored images were measured to determine the velocity of the fastest moving group of particles using NIH ImageJ software (http://rsb.info.nih.gov./ij/) and MCCV was expressed in mm/min. Our MCC assays were done over a 1.5-year period, with each set of like experiments done together on 1–3 batches of ferret tracheas (~6 per batch). Variation of basal and stimulated MCCV within groups of experiments was less than variation across groups of experiments. Therefore, the most meaningful data are for the paired control and drug-treated tracheas within the chamber at the same time, as shown, for example, in Fig. 3C–F.

### Protocols for drug treatments

Each trachea was treated with either a CFTR inhibitor (20 μM CFTR_inh_-172 or an ENaC inhibitor 10 μM benzamil) or vehicle (0.1% DMSO was used in all control baths) applied to both the apical surface (1 ml of the drug in KRB buffer) and bath (~3 ml) for 30 min at 37 °C in the sealed MCC device. After 30 min of an inhibitor or control treatment, solutions were drained from the surfaces and the baths of the tissue preparations and the baths were refreshed with pre-warmed KRB ± an inhibitor (with DMSO in the control bath). The tracheas were then equilibrated for 15 min which allowed any excess fluid to be cleared from the apical surface (Fig. 1B). The bath was then refreshed with pre-warmed KRB containing an agonist (10 μM forskolin, 10 μM isoproterenol or 0.3 μM carbachol) ± an inhibitor. Basal MCCV was measured between T0-T1 before the onset of agonist-induced increases in MCCV. Responses to treatments were measured at 5 min intervals as described. To assess synergy, after 30 min (60 in a few experiments) the second agonist was added and MCCV followed for an additional 30 min. In some experiments, we used 20 μM CFTR_inh_-172 + 50 μM GlyH101 and 0.2% DMSO as a vehicle control.

### Reagents

The compounds used in the present study were purchased either from Sigma-Aldrich (St. Louis, MO) or Calbiochem (Billerica, MA) and were prepared fresh or maintained at −20 °C as aliquots of stock concentration. Benzamil, forskolin, CFTR_inh_-172, and GlyH101 were dissolved in dimethyl sulfoxide (DMSO), carbachol and isoproterenol were dissolved in sterile double distilled water, and indomethacin was dissolved in absolute ethanol. All chemicals were diluted 1: 1,000 with bath KRB buffer (except indomethacin, 1: 10,000) immediately before use at the concentrations indicated.

### Statistics

Data are presented as mean ± S.E.M. unless indicated otherwise. To determine if the data were normally distributed, we ran a normality test (a cumulative distribution plot) on our synergy data where n = 90, and found that the actual distribution curve almost overlapped with the normal distribution one. Given this indication of normally distributed data, we used unpaired, two-tailed Student’s t-test to compare means of different treatment groups. Differences were considered to be significant when P < 0.05. We also performed Mann-Whitney U tests when Student t-test P values were between 0.01 and 0.05; P values changed only slightly and in no case did the conclusion as to significance change.

## Additional Information

**How to cite this article**: Joo, N. S. *et al*. Marked increases in mucociliary clearance produced by synergistic secretory agonists or inhibition of the epithelial sodium channel. *Sci. Rep*. **6**, 36806; doi: 10.1038/srep36806 (2016).

**Publisher’s note:** Springer Nature remains neutral with regard to jurisdictional claims in published maps and institutional affiliations.

## Supplementary Material

Supplementary Information

Supplementary Video S1
