# Supplementary material for: Marked increases in mucociliary clearance produced by synergistic secretory agonists or inhibition of the epithelial sodium channel
Source: Sci Rep. 2016 Nov 10;6:36806. doi: 10.1038/srep36806 (PMC5103292; doi:10.1038/srep36806)
Supplement: Supplementary Information [file srep36806-s1.pdf]

# Marked increases in mucociliary clearance produced by synergistic secretory agonists or inhibition of the epithelial sodium channel

**Nam Soo Joo[1*], Jin Hyeok Jeong[1,2], Hyung-Ju Cho[1,3], and Jeffrey J. Wine[1*]**

**Supplementary Figure S.1**

**Supplementary MCC video legend**

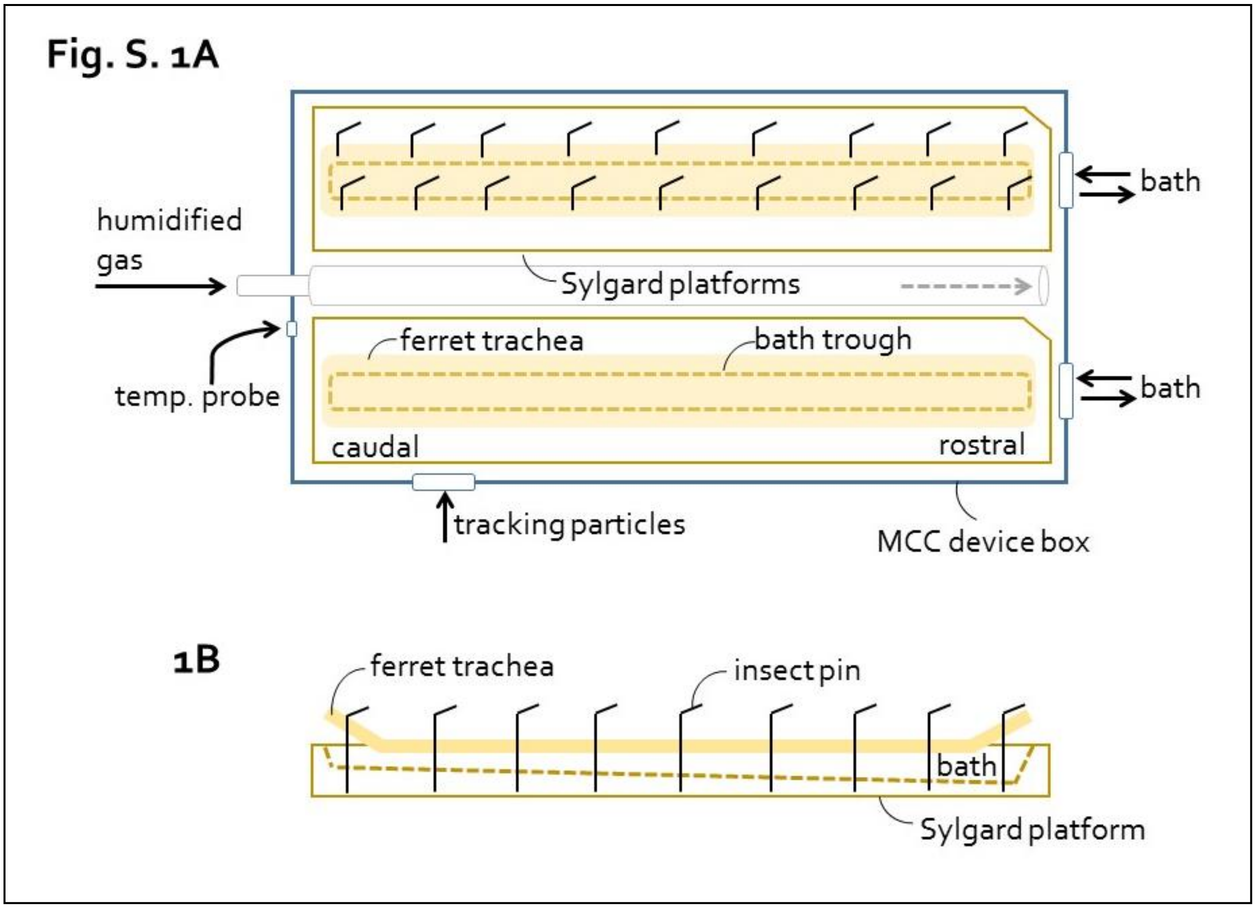


**Supplementary Figure S. 1. A modified MCC device schematic.** (**A**) ***Top view*** of the MCC device box showing: two ferret tracheal preparations mounted on Sylgard platforms using insect pins; a central inlet of humidified 95%/5%-$O_2/CO_2$ designed to reach the opposite side (rostral) to minimize a potential unequal/direct impact of humidity on the caudal portions of the tracheae; and openings for bath exchange and for a temperature probe. (**B**) ***Side view*** of a Sylgard platform and a mounted trachea. The angled trough of a Sylgard platform was designed to facilitate bath exchange during MCC assays and both caudal and rostral ends of each tracheal preparation were mounted slightly upward to prevent a potential entry of bath fluid onto the surface epithelia.

Legend for Supplementary MCC video

**Increased mucociliary clearance by ENaC inhibition and by the synergy paradigm.** A representative video shows mucociliary clearance of two tracheal preparations induced by 10 μM forskolin, top one as a control and bottom one with prior apical and basolateral 10 μM benzamil treatment for 30 min and continuous presence of benzamil at the bath. The movie is comprised of 10 μM Fsk ± 10 μM Bz for 30 min, followed by 10 μM Fsk + 0.3 μM carbachol for additional 30 min in the absence of benzamil. The images were taken T1, and every 5 min intervals and 4 images/min (0, 20, 40, and 60 second). Note that enhanced movement of particles at T0-T1 and the rest of Fsk + Bz period at the bottom trachea and synergistic MCC induced by the combined agonists in both tracheas at the 2nd half of the movie.
